# Impact of highly active antiretroviral therapy on hematological indices among HIV-1 infected children at Kenyatta National Hospital-Kenya: retrospective study

**DOI:** 10.1186/s12981-015-0069-4

**Published:** 2015-08-15

**Authors:** Elizabeth Gathoni Kibaru, Ruth Nduati, Dalton Wamalwa, Nyambura Kariuki

**Affiliations:** Department of Paediatrics and Child Health, Egerton University, P.O. Box 536-20115, Egerton, Kenya; Department of Paediatrics and Child Health, University of Nairobi, P. O. Box 19676-00202, Nairobi, Kenya; Paediatric Hematology and Oncology, University of London, London, UK; P.O. Box 2275-20100, Nakuru, Kenya

**Keywords:** Paediatric HIV infection, Hematological abnormalities, Changes of hematological parameters

## Abstract

**Background:**

HIV infected children experience a range of hematological complications which show marked improvement within 6 months of initiating anti-retroviral therapy. The Objectives of the study was to describe the changes in hematological indices of HIV-1 infected children following 6 months of treatment with first line antiretroviral drugs (ARVs) regimen.

**Methods:**

A retrospective study was conducted between September and November 2008. During this period medical records of children attending Comprehensive Care Clinic at Kenyatta National hospital were reviewed daily. HIV infected children aged 5–144 months were enrolled if they had received antiretroviral drugs for at least 6 months with available and complete laboratory results.

**Results:**

Medical records of 337 children meeting enrollment criteria were included in the study. The median age was 63 months with equal male to female ratio. Following 6 months of HAART, prevalence of anemia (Hemoglobin (Hb) <10 g/dl) declined significantly from 35.9 to 16.6 % a nearly 50 % reduction in the risk of anemia RR = 0.56 [(95 % CI 0.44, 0.70) p < 0.001]. There was significant increase in Hb, mean corpuscular volume (MCV), mean corpuscular hemoglobin (MCH) and platelets above the baseline measurements (p < 0.0001) and a significant decline in total white blood cell counts >11,000 cell/mm^3^ but a none significant decrease in red blood cells (RBC). Pre-HAART, World Health Organization (WHO) stage 3 and 4 was associated with a ten-fold increased likelihood of anemia. Chronic malnutrition was associated with anemia but not wasting and immunologic staging of disease.

**Conclusion:**

Hematological abnormalities changed significantly within 6 months of antiretroviral therapy with significant increase in hemoglobin level, MCV, MCH and platelet and decrease in WBC and RBC.

## Background

Hematological complications of human deficiency virus infection (HIV) which include cytopenias of all major cell lines were recognized shortly after the first description of AIDS cases [1]. Unexplained anemia defined as hemoglobin of less than 8 g/dl, neutropenia <1000 cells/mm^3^ or thrombocytopenia less than 30,000 platelets/mm^3^ persisting for more than one month are currently classified as WHO stage 3 disease [2]. Anemia, the most common abnormality causes chronic fatigue, affects cognitive function and influences the choice of ART and opportunistic infection (OI) medications and has been noted to be the commonest hematological abnormalities in adults in Kenya [3, 4].

In published literature prevalence of anemia among HIV-1 infected children varies from 16 to 94 % and this increases with advancing stage of HIV disease but varies with sex, age and the definition of anemia used [5–10]. In a meta-analysis of over 2000 HIV infected children, the prevalence of mild [hemoglobin (Hb) 10–12.0] and moderate anemia (Hb 8.0–9.9) was 22–94 % and 3–82 % respectively [10]. The prevalence of mild and moderate anemia in Western settings ranged from 22 to 94 % and 11 to 82 % respectively while in tropical settings it was 50–91 % for mild anemia and 3–38 % for moderate anemia [10]. Neutropenia is present in approximately 10 % of patients with early, asymptomatic HIV infection, and more than half in individuals with more advanced HIV-related immunodeficiency [5, 6, 11]. Thrombocytopenia which is frequently asymptomatic occurs in 20 % to 33 % of paediatric patients with HIV with the prevalence increasing with duration of illness and development of Aids [5, 6, 9, 11, 12].

The most important cause of anemia in HIV infected children is insufficient production of erythrocytes caused by ineffective erythropoiesis, reduced erythropoietin production, associated infections, neoplasia, medications and micronutrient deficiencies. Iron deficiency is the most common cause of nutritional anemia globally. There is significant geographical overlap of areas of the world where iron deficiency anemia (IDA) and paediatric HIV are distributed. The causes of anemia in the community investigated are Multifactorial with iron deficiency prevalence being high due to nutritional deficiency and helminthes. In a study in western Kenya by Footer et al. anemia was found to be prevalent and was present in 71.8 % of children [13]. Factors commonly thought to be associated with anemia were found to be widespread, including malaria parasitemia (32.5 %), iron deficiency (34.6 %), vitamin A deficiency (16.3 %), stunting (29.6 %), wasting (3.3 %), HbS (17.1 %), HbSS (1.6 %), heterozygous α-thalassemia (38.5 %), and homozygous α-thalassemia (9.6 %) [13]. This is similar to WHO finding that Iron deficiency is the most common cause of anemia of micronutrient-related etiology worldwide, affecting about 50 % of women and children in developing countries and about 25 % of men [14]. Another prominent cause of reduced production of all hematological elements in patients with HIV infection is the common use of multiple medications, many of which may cause marrow suppression e.g. Zidovudine (AZT) and other nucleoside analogues can cause a dose-dependent neutropenia [15]. Other medications causing hematological abnormalities include ganciclovir, trimethoprim-sulphamethoxazole, pentamidine and dapsone [9].

Studies have confirmed the ability of HAART to correct or improve the anemia and other hematological parameters of HIV infection. In a study of 6725 HIV-infected patients from across Europe, Microft and colleagues found that the use of Stavudine, zidovudine and Lamivudine was statistically associated with improvement in hemoglobin levels [16]. When used for longer periods of time, HAART was associated with greater likelihood of correcting anemia. Microft et al. found that, 65.5 % of patients were anemic before the use of HAART, 53 % were anemic after 6 months of HAART (p < 0.0001), and 46 % were anemic after 12 months of HAART [16]. Huang et al. found an increase in Hb occurring with mean increase from baseline of 13.9 to 14.1 g/dl after 3 months of treatment and this was not statistically significant [17]. After 6 months Hb had increased to 14.6 g/dl (p < 0.01), 9 months 14.6 g/dl (p = 0.001) and at 12 months mean of 14.3 g/dl (p < 0.01) [17]. In a different study in children in Zambia, the mean hemoglobin increased from 10.3 g/dl at baseline to a mean of 11.3 g/dl (CI 95 % 11.2–11.4 g/dl) after 6 months of HAART [18]. In a study in Burkina-Faso hematological changes after 6 months of AZT based HAART showed anemia at 51.4 vs 80.3 % at baseline with P = 0.0001 [19]. Other hematological parameter noted to markedly increase after HAART was MCV. Huang et al. found an increase of MCV following treatment from baseline of 5.5 to 98.9 fl p < 0.1 at 3 months, 105.5 fl (p < 0.001) at 6 months, 106 fl at 9 months (p < 0.001) and 102.8 fl p < 0.001 at 12 months [17].

The prevalence of hematological abnormalities among children treated with ART in routine care settings in Kenya have not yet been described even though the first line antiretroviral regimen to date includes drugs with significant hematological adverse events such as zidovudine. The objective of the study was to describe the changes in hemoglobin levels, red cell morphology, white blood cells and platelets in HIV-1 infected children aged 5–144 months following 6 months of highly active antiretroviral therapy at the comprehensive care clinic of KNH.

## Results

### Baseline characteristics of the study participants

A total of 337 children were recruited into the study, among them 53.4 % female and 46.6 % male giving a female to male ratio of 1.1:1, and a median age of 63 months (range of 5–144 months) as shown in the Table 1. Almost all children in the study population received Cotrimoxazole 323 (98.8 %) and the remaining 4 (1.2 %) received dapsone. One eighty nine (56.4 %) presented in WHO clinical stage III at ART initiation while 48 (14.3 %) were in WHO clinical stage IV. The number/proportion of children in stages I and II were 77 (23 %) and 21(6.3 %) respectively. Eighty four children (25 %) were started on iron supplements during the initiation of antiretroviral drugs. Zidovudine-Lamivudine backbone (AZT/3TC) based regimens were the most frequently used first line ART protocol and used by 291 (86.3 %) children in combination with efavirenz (EFV), nevirapine (NVP) or abacavir (ABC).

**Table 1:** Baseline demographic characteristics of the study participants

Variable	Frequency (n = 337) number, (%), or median
Female	179 (53.4 %)
Male	156 (46.6 %)
Age (months)	
Median	63
IQR	36–97
Weight for age Z score	
SD > −2	130 (39 %)
SD −3 to −2	95 (28 %)
SD < −3	112 (33 %)
Weight for height Z score	
SD > −2	229 (88 %)
SD −3 to −2	22 (6.5 %)
SD < −3	15 (4.4 %)
Height for age Z score	
SD > −2	85 (25 %)
SD −3 to −2	53 (16 %)
SD < −3	198 (59 %)
CD4 count median (1QR)	456 (171.0–753)
CD4 percent median (1QR)	9.9 (5.0–14.0)

### Hematological parameters pre and six months post HAART initiation

#### Hemoglobin level

At baseline 35.9 % study participants had a Hb <10.0 g/dl compared to 16.6 % after 6 months of ART, a statistically significant reduction in risk of anemia RR = 0.56 [(95 % CI 0.44, 077) p < 0.0001] as shown in Table 2. The median Hb at baseline was 10.6 and 11.5 g/dl before and after 6 months of ART, a significant median increase in Hb of 0.9 g/dl (p < 0.0001) as shown in Table 3. The median Hb level of 203 (75 %) children who were not on iron supplements at baseline and after 6 months of ART was 11 g/dl, and 11.8 g/dl respectively a statistical significant change (p < 0.0001). Eighty four children (25 %) who were receiving iron supplements had a baseline median Hb of 9.25 g/dl and increased to 11 g/dl following 6 months of treatment, a significant median increase of Hb 1.75 g/dl (p < 0.0001). Female were reported to have higher hemoglobin levels at the baseline 51.9 % compared to the males 40.3 % although there were more males with moderate anemia (37 %) compared to female 23.1 %. Following 6 months of HAART majority of children of both sexes had hemoglobin levels within the normal ranges with 73.7 % being female and 68.5 % males. Children who initially were reported with severe anemia had reduced to 1.3 % from 4.5 % of female with no male reported to have severe anemia.

**Table 2:** Changes in the prevalence of abnormal haematological parameters

Characteristic N = 337	Baseline no (%)	6 months after initiation of HAART no (%)	Relative risk (95 % CI)	P value
Hb <10 g/dl	121 (35.9)	56 (16.6)	0.56 (0.44, 0.70)	<0.0001
MCV <70 fl	104 (31.0)	28 (8.3)	0.37 (0.27, 0.52)	<0.0001
MCH <24 pg	154 (46.7)	49 (14.6)	0.45 (0.35, 0.59)	<0.0001
Platelets <150,000	67 (20.0)	22 (6.5)	0.46 (0.32, 0.66)	<0.0001
WBC <11,000	33 (10.0)	21 (6.2)	0.76 (0.54, 1.08)	0.09
Granulocytes <1000	22 (6.5)	52 (16)	1.48 (1.25, 1.75)	<0.0001

**Table 3:** Changes in haemoglobin levels, MCV, MCH and RBC

Characteristic N = 337	Baseline median no (%) or (IQR)	6 months after initiation of HAART no (IQR)	Median change	P value
Hb (g/dl)	10.6 (9.4–11.6)	11.5 (10.5–12.4)	0.9	<0.0001
MCV (fl)	75.9 (68.0–82.3)	89.2 (80.0–97.6)	13.2	<0.0001
MCH (pg)	24.3 (21.5–27.5)	29.1 (25.8–32.1)	4.9	<0.0001
RBC (m/mm^3^)	4.3 (3.8–5.0)	4.0 (3.6–4.3)	0.3	0.27

#### Mean corpuscular volume (MCV)

At baseline 31 % of the study participants had a mean corpuscular volume (MCV) below the 70 fl compared to 8.3 % at end of 6 months of ART treatment, a 63 % decline in the risk RR = 0.37 [(95 % CI 0.27,0.52) P < 0.0001] as shown in Table 2. The median corpuscular volume at baseline was 75.9 fl and increased to 89.2 fl after 6 months of HAART, a statistically significant median increase by 13.2 fl of corpuscular volume (p < 0.0001) as show in Table 3.

#### Mean corpuscular hemoglobin (MCH)

The prevalence of low mean corpuscular hemoglobin (MCH) before and after 6 months of ARVs was 46.7  and 14.6 % respectively. The relative risk of low MCH was RR = 0.45 [(95 % CI 0.35, 0.59) p < 0.0001], a statistically significant reduction. At baseline median MCH was 24.3 pg and increased to 29.0 pg after 6 months of ART a statistically significant median increase of 4.9 pg (p < 0.0001) as shown in Table 3.

#### Red blood cells

At baseline the median number of red cells was 4.3 million/mm^3^ and declined to 4.0 million/mm^3^ after 6 months of highly active antiretroviral therapy. The median change in RBC was 0.4 million cells/mm^3^ and this was not statistically significant (p = 0.27) as shown in Table 3.

#### HAART regimen combination and hemoglobin level

Children with Stavudine combination had higher hemoglobin increments as compared to zidovudine or other combinations as shown in Table 4. There was statistical significant increase in mean Hb level in both zidovudine and Stavudine combinations but the mean hemoglobin change was more with Stavudine combinations at 1.83 g/dl for Stavudine combination and 0.7 g/dl for zidovudine combinations. Indications of choice of regimen to use were based on baseline hemoglobin levels. Majority of children with Hb of above10 g/dl were started on zidovudine combination while Children with low Hb of less than 10 g/dl were started on Stavudine or other combinations.

**Table 4:** Haemoglobin level changes and HAART combination

Drug regimen	Number	Mean Hb at baseline	Mean Hb after 6 months	Mean change	*P* Value
Zidovudine combination	293	10.7	11.4	0.7	0.000
Stavudine combination	38	9.77	11.6	1.83	0.01
Other combinations	6	7.68	11.2	3.52	0.864

#### Platelets

**Fig. 1:**
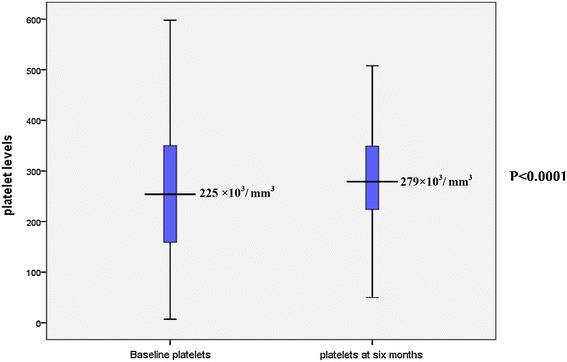
Comparison of platelet levels at baseline and after 6 months of treatment

Proportion of children with platelet counts below 150,000/mm3 before and after 6 months of ARV was 20 and 6.5 % respectively, a significant reduction in the risk of thrombocytopenia RR = 0.46 [(95 % CI 0.32, 0.66) p < 0.0001] as shown in Table 3. At baseline the median platelets counts were 255 × 10^3^/mm^3^ (IQR 159 × 10^3^/mm^3^–352 × 10^3^/mm^3^), and which increased to a median of 279 × 10^3^/mm^3^ (IQR 224 × 10^3^/mm^3^–349 × 10^3^/mm^3^) following 6 months of ART as shown in Fig. 1.

#### White blood cells

Before ART initiation 10 % of the children had elevated white blood cell counts above 11,000 cell/mm^3^ compared to 6.2 % after 6 months of ARV initiation. The proportion of children with elevated total WBC thus decreased with ART RR = 0.76 [(0.54, 1.08) P = 0.09] a non-significant decline. Overall the median total WBC count at baseline was 9.4 × 10^3^/mm^3^ which declined significantly to a median of 6.7 × 10^3^ cells/mm^3^ (p < 0.0001). At baseline only 6.5 % of the study participants had granulocyte counts of less than 1000 cells/mm^3^ compared to 16 % after 6 months of ARVs, a statistically significant increased likelihood of having low granulocyte counts RR = 1.48 [(1.25, 1.75), P < 0.0001]. The total granulocyte count declined significantly following 6 months of ART from a median of 2.8 × 10^3^/mm^3^–2 × 10^3^/mm^3^ (IQR 1.4 × 10^3^/mm^3^–5.1 × 10^3^/mm^3^) (p < 0.0001) as shown in Table 5. The median lymphocyte count was of 5.2 × 10^3^ cell/mm^3^ before treatment initiation and declined significantly to 4.2 × 10^3^ cell/mm^3^, (p < 0.0001) following 6 months of ART.

**Table 5:** Changes in WBC, Granulocytes and lymphocytes following six months of HAART

Characteristic N = 337	Baseline Median (IQR)	6 months after of HAART Median (IQR)	Median change	P value
WBC (×10^3^/mm^3^)	8.7 (IQR 6.2–11.5)	6.7 (IQR 5.2–8.5)	2.0	<0.0001
Granulocytes × 10^3^/mm^3^	2.8 (IQR 1.8–4.3)	2.0 (IQR 1.4–2.9)	0.8	<0.0001
Lymphocytes × 10^3^/mm^3^	4.8 (2.9–6.7)	4.2 (3.0–5.1)	0.6	<0.0001

### Factors associated with low baseline hemoglobin level

In a stratified analysis the 216 (64.1 %) and 121 (35.9 %) children with Hb above and below 10 g/dl respectively were compared to determine demographic and clinical characteristics associated with anemia. Children in WHO stage 3 and 4 were more likely to have Hb of less than or equal to 10 g/dl compared to children in WHO stage 1 and 2 OR = 10.6 (95 % CI 3.64, 36.3) (P < 0.001). Children with WAZ greater than −2 SD had significantly reduced likelihood of having Hb of less than 10g/dl OR = 0.62 [(95 % CI 0.38, 1.01) P = 0.04] as shown in Table 6.

**Table 6:** Correlates of low baseline haemoglobin level

	Hb ≥ 10 N = 216 (64.1 %)	Hb ≤10 N = 121 (35.9 %)		P value
CD4 (median)	9.0	10.8		0.2
WHO				<0.0001
Stage 1 and 2	76 (35.2 %)	4 (8.2 %)	1	
Stage 3 and 4	140 (64.8 %)	80 (81.8 %)	10.6 (3.64, 36.3)	
WHZ				0.58
<−2	26 (12.0 %)	17 (14.2 %)	1	
>−2	190 (88.0 %)	103 (85.8 %)	0.83 (0.41, 1.68)	
WAZ				0.04
<−2	124 (57.4 %)	83 (68.6 %)	1	
>−2	92 (42.6 %)	38 (31.4 %)	0.62 (0.38, 1.01)	

## Discussion

In this study we report increase in most hematological indices following 6 months of ART in children. Following 6 months of treatment with HAART, hematological reconstitution occurred progressively for all blood lineages except RBC, total WBC, granulocytes and total lymphocytes counts. The positive effect of HAART is probably due to the reduction in viral load, decreased destruction of mature hematopoietic cells of multiple lineages and an improvement in the blunted erythropoietin response [11]. HAART could also have lead to decreased incidences of opportunistic infections [17].

The median increase in Hb levels of 0.9 g/dl was well within the range of the 0.8–1.0 g/dl in published studies. Microft et al. in Europe and Huang et al. reported an increase from 13.9 to 14.6 g/dl a statistical association between the level of Hb and duration of use of HAART was also found [16, 17]. In children, Bolton-Moore et al. found that for children with baseline mean Hb of 10.3 g/dl, it had increased to 11.3 g/dl after6 months of HAART almost similar to our study [18]. In another study in Burkina-Faso, Nacoulma et al. found that after 6 months of AZT based HAART anemia prevalence had reduced [19]. Drug regimen was noted to have a big influence on the increments in hemoglobin level with Stavudine combination having a much higher increment as compared to zidovudine combination findings similar to other studies [20].

In this study children who received iron supplements were noted to have higher hemoglobin increments this is similar to the findings in Malawi where iron supplementation in HIV infected children increased hemoglobin levels and reduced the prevalence of anemia by 40 % but iron was noted to increase the risk of malaria [21]. Identification of children to be given iron in this study was based on low baseline hemoglobin levels as recommended by WHO on targeted iron supplementation for children at risk of anemia and iron deficient [14]. Children who received these drugs had markedly reduced hemoglobin level at baseline. Some of these children were also provided with food supplement and nutritional education and this could have contributed to marked increase. The study was not able to identify whether those given iron supplements had higher risk of sepsis due to retrospective nature of the study.

When we compared the mean corpuscular volume at baseline of 75.9 fl with that after 6 months of treatment of 89.2 fl, we found an increase in median change that was higher than the findings of Huang et al. in adults of 95.5 fl at baseline to 105.5 fl following 6 months of HAART [17]. The increase could have been due to nutritional modification offered, iron supplements or antiretroviral drugs. Macrocytosis was found to have occurred after treatment and this could be attributed to the effect of mainly zidovudine as this has been found in other studies [22]. A striking and discrepant observation was that the circulating RBC counts decreased significantly following six months of HAART despite increase in hemoglobin levels and mean corpuscular volume. This finding is similar to the findings of Huang et al. and may be associated with defect in the production of erythrocytes from erythroid progenitor cells leading to the generation of fewer but larger cells [17].

Total white blood cells were found to decrease significantly from 8.7 × 10^3^ mm^3^ to 6.7 × 10^3^ mm^3^ in this study probably because at the time of enrollment, the children might have had intercurrent acute or chronic infections causing leucocytosis and following treatment and initiation of HAART the levels were seen to have decreased and this could have been due to the antiretroviral drugs improving immunity leading to less infections. Similar findings were reported by Nacoulma et al. in adults after six months of AZT based HAART of 2.27 × 10^3^ mm^3^ at baseline compared to 1.9 × 10^3^ mm^3^ after 6 months of HAART [19]. The finding in this study contrast some studies which reported significant increase in WBC following treatment with Huang et al. reporting a mean increase of 0.8 (p < 0.001) [17]. In this study, there was a statistical significant increase in platelets levels from 255 × 10^3^ mm^3^ to 279 × 10^3^ mm^3^ similar to findings by Huang et al. in adults [17].

In this study the first line regimens as recommended by the Ministry of Health of both zidovudine based and Stavudine based were shown to lead to a significant change in hematological parameters similar to the findings in various other studies [17, 18]. Moyle G et al. in their study also assessed the mean decreased in hemoglobin level comparing various regimens after treatment which we were unable to do due the small number of patients that were started on Stavudine based and protease inhibitor based regimens [4].

## Conclusion

The findings of this study suggest that Antiretroviral drugs lead to a significant change in hematological parameters with a positive change of hemoglobin levels, MCV, MCH and platelet and decrease in total WBC, granulocytes lymphocytes and RBC. This indicates that zidovudine based regimens may safely be used even in setting with limited laboratory monitoring.

## Methods

This retrospective cohort study was carried out at Kenyatta National Hospital comprehensive care centre between September and November 2008. Children were enrolled into the study if they were age 5–144 months, confirmed to be HIV positive by rapid tests for those aged over 18 months and PCR DNA for those aged less than 18 months, attending the Kenyatta National Hospital comprehensive care center. They also had to have received the first line ART according to the Kenyan Ministry of Health guidelines without change of treatment for at least six months with available complete medical records and parents/guardians provided informed consent. Standard study tools were used to abstract data from the medical records of enrolled children and included demographic, clinical and laboratory characteristics. The laboratory data had been analyzed by University of Nairobi Paediatric laboratory which undertakes laboratory procedures relating to the comprehensive care clinic. It uses a semi automated hematology analyzer MS4 and is a GCLP certified (Good Clinical Laboratory Practices) with external quality assurance. There is computerized data for all children attending the clinic who are on HAART and it also details of the regimen the children is on and when started. Using ART number in the data base and patients appointment number we were able to identify the patients’ files. Files were retrieved as the clients came for review in their monthly clinic that runs daily from Monday to Friday. Pre and 6-month post ART estimates of, absolute CD4, CD4%, hemoglobin level, total white blood cells count, absolute lymphocyte counts, granulocyte counts, and platelets counts were recorded in an abstract form. In addition information was obtained regarding use of all medications including prophylactic medications, iron supplementation, multivitamins, and HAART regimens. Ethical clearance to conduct the study was obtained from the department of Pediatrics and Child Health and the KNH ethical review Committee.

### Statistical methods

Data entry and analysis were done using SPSS version 16.0. The weight for height Z scores (WHZ), height for age Z scores (HAZ) and weight for age Z scores (WAZ) were computed using the nutrition software of Epi Info 3.2 Baseline hematological parameters were compared to those obtained after 6 months of HAART using Pearson Chi square test to compare categorical variables. Wilcoxon paired t-test for non parametric variables was used to determine the difference in baseline hematological parameters and parameters after 6 months of HAART for parameters. Differences in associations and relationships were taken as significant where P was less than 0.05.

## Abbreviations

HIV: human immunodeficiency virus
HAART: highly active antiretroviral therapy
ARV: anti retroviral therapy
KNH: Kenyatta National Hospital
Hb: hemoglobin level
RR: relative risk
MCV: mean corpuscular volume
MCH: mean corpuscular hemoglobin
WHO: World Health Organization
WBC: white blood cells
OI: opportunistic infection
ART: anti retroviral therapy
IDA: iron deficiency anemia
AZT: zidovudine
NVP: nevirapine
EFV: efavirenz
RBC: red blood cells
PCR: polymerase chain reaction
ABC: abacavir
3TC: Stavudine
GCLP certified: good clinical laboratory practices
